# Supplementary material for: Large library docking for cannabinoid-1 receptor agonists with reduced side effects
Source: bioRxiv. 2024 Jan 23:2023.02.27.530254. Preprint. [Version 2] doi: 10.1101/2023.02.27.530254 (PMC10849508; doi:10.1101/2023.02.27.530254)
Supplement: Supplement 1 [file media-1.pdf]

Supplementary Information for

Large library docking for cannabinoid-1 receptor agonists with reduced side effects

Tia A. Tummino[1,2,]†, Christos Iliopoulos-Tsoutsouvas[3,]†, Joao M. Braz[4,]†, Evan S. O'Brien[5], Reed M. Stein[1,2], Veronica Craik[4], Ngan K. Tran[3], Suthakar Ganapathy[3], Fangyu Liu[1], Yuki Shiimura[5,6], Fei Tong[3], Thanh C. Ho[3], Dmytro S. Radchenko[7], Yurii S. Moroz[7,8,9], Sian Rodriguez Rosado[4], Karnika Bhardwaj[4], Jorge Benitez[4], Yongfeng Liu[10], Herthana Kandasamy[11], Claire Normand[11], Meriem Semache[11], Laurent Sabbagh[11], Isabella Glenn[1], John J. Irwin[1], Kaavya Krishna Kumar[5,]*, Alexandros Makriyannis[3,12]*, Allan I. Basbaum[4,]*, & Brian K. Shoichet[1,*,#]

†Contributed equally.

[#]Lead Contact

*Correspondence: kaavyak@stanford.edu, a.makriyannis@northeastern.edu, allan.basbaum@ucsf.edu, bshoichet@gmail.com

This file includes:

Supplementary Tables 1 to 8

Additional supplementary materials:

Synthetic procedures, chemical characterization and spectral data is supplied as
Supplemental_Information_QC.pdf
Supplementary_Table_9.xlsx
Model_final.pdb
Map_final.mrc
PDB_Validation_report.pdf

Supplementary Table 1. Binding affinities for hits identified in initial CB1 docking screen.

Compound	Global rank	rCB1 affinity[a]<br>K_i [95% CI] nM<br>pK_i [95% CI]	Tc[b]	Nearest ChEMBL ligand[c]
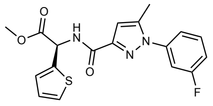<br>‘51486	117390	731 [552 – 969]<br>6.14 [6.01 –6.26]	0.30	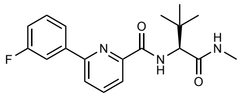<br>CHEMBL4110127
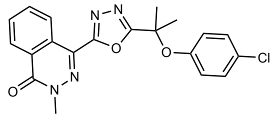<br>‘0450	6582	691 [459 – 1033]<br>6.16 [5.99 – 6.34]	0.36	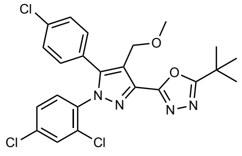<br>CHEMBL519214
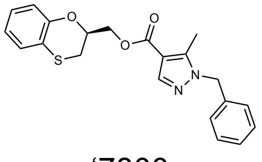<br>‘7800	12210	1007 [615 – 1654]<br>6.0 [5.78 – 6.21]	0.28	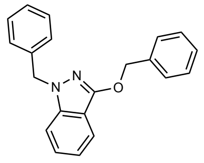<br>CHEMBL3116279
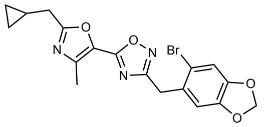<br>‘7019	20488	4039 [3027 – 5379]<br>5.39 [5.27 –5.52]	0.24	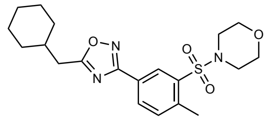<br>CHEMBL472680
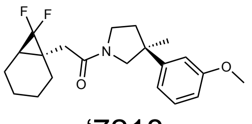<br>‘7218	29322	52.2% [24.79]	0.31	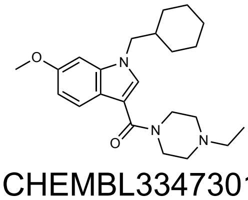<br>CHEMBL3347301
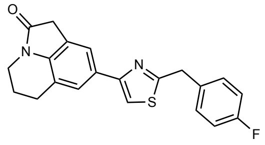<br>‘1038	47606	53.6% [2.91]	0.28	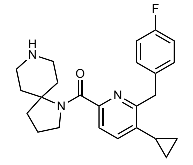<br>CHEMBL3890211
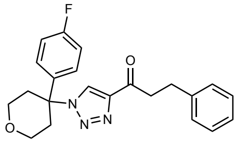<br>‘7337	24720	57.0% [3.04]	0.29	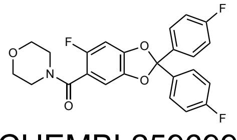<br>CHEMBL259699
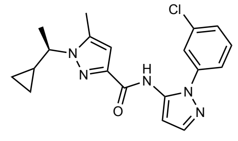<br>‘7902	139929	57.1% [0.02]	0.31	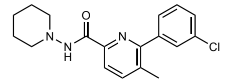<br>CHEMBL3915046

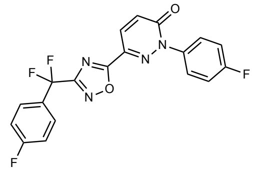 '2443	21964	51.1% [4.87]	0.23	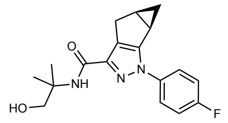 CHEMBL3354970

[a]Binding affinity to rCB1 represented as Ki [95% CI] and pKi [95% CI] from three independent experiments in triplicate when measured. Otherwise, % radioligand displacement [S.D] from three replicates in a single-point competition experiment at 10 μM

[b]Tanimoto coefficient based on ECFP4 fingerprints

[c]Corresponding ChEMBL ligand with the most similar fingerprint

Supplementary Table 2. **Binding affinities and functional activities for active analogs at CB1.**

Compound	rCB1 binding<br>K_i [95% CI] (nM)<br>pK_i [95% CI]<br>E_{max} [SEM]<br>Significance[a]	hCB1 Lance Ultra cAMP<br>EC_{50} [95% CI] (nM)<br>pEC_{50} [95% CI]<br>E_{max} [95% CI]<br>Significance[a]	hCB1 Cerep cAMP<br>EC_{50} [95% CI] (nM)<br>pEC_{50} [95% CI]<br>E_{max} [95% CI]	hCB1 Glosensor cAMP<br>EC_{50} [95% CI] (nM)<br>pEC_{50} [95% CI]<br>E_{max} [95% CI]	hCB1 Tango ß-arrestin recruitment<br>EC_{50} [95% CI] (nM)<br>pEC_{50} [95% CI]<br>E_{max} [95% CI]	hCB1 DiscoverX ß-arrestin recruitment<br>EC_{50} [95% CI] (nM)<br>pEC_{50} [95% CI]<br>E_{max} [95% CI]
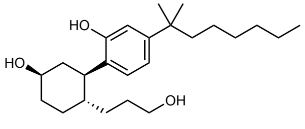<br>**CP–55,940**	2.9 [2.05 – 4.2]<br>8.5 [8.4 – 8.7]<br>98% [3.5]	6.2 [4.7 – 8.0]<br>8.2 [8.1 – 8.3]<br>85% [86 – 85]	0.026	0.028 [0.02 – 0.04]<br>10.6 [10.5 – 10.7]<br>96% [93 – 99]	8.9 [7.5 – 10.6]<br>8.1 [8.0 – 8.1]<br>100% [96 – 104]	4.0 [3.2 – 4.9]<br>8.4 [8.3 – 8.5]<br>108% [99 – 109]
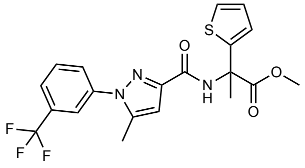<br>**‘4042**	1.86 [1.37 – 2.52]<br>8.7 [8.6 – 8.9]<br>99% [3.0]<br>ns	3.3 [1.9 – 5.6]<br>8.5 [8.3 – 8.7]<br>78% [78 – 79]<br>ns	0.008 [0.006 – 0.01]<br>11.1 [11.0 – 11.2]<br>96% [102 – 107]	0.039 [2.9 – 5.4]<br>10.4 [10.3 – 10.5]<br>91% [87 – 94]	10.7 [8.7 – 13.3]<br>8.0 [7.9 – 8.1]<br>102% [98 – 105]	2.3 [2.5 –4.8]<br>8.7 [8.3 – 9.6]<br>71% [60 – 65]
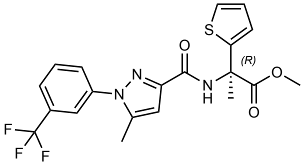<br>**‘1350**	0.95[.74 – 1.24]<br>9.02 [8.9 – 9.1]<br>106% [2.9]<br>*	1.6 [0.7 – 3.6]<br>8.8 [8.4 – 9.2]<br>78% [77 – 80]<br>**	--	--	--	--
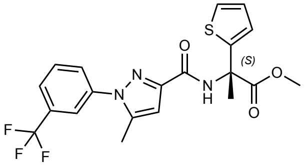<br>**‘8690**	90.2 [56.7 – 143]<br>7.1 [6.9 – 7.3]<br>100% [4.0]<br>****	473 [109 – 1822]<br>6.3 [5.8 – 7.0]<br>53% [45 – 65]<br>****	--	--	--	--

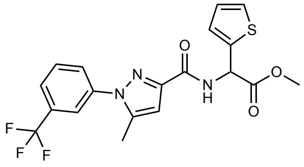 **‘60154**	44.3 [33.9 – 58.0]<br>7.4 [7.2 – 7.5]<br>110% [2.9]	351 [93]<br>6.5 [7.0]<br>67% [59 – 89]	--	25.2 [16 – 40]<br>7.6 [7.4 – 7.8]<br>82% [74 – 189]	819 [718 – 934]<br>6.1 [6.0 – 6.1]<br>39% [37 – 40]	--
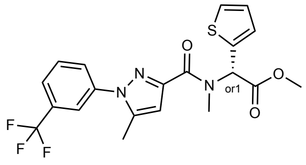 **‘1066**	1719 [736 – 4048]<br>5.8 [5.4 – 6.1]<br>97% [8.0]					
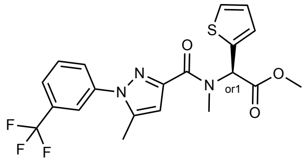 **‘6000**	1455 [943 – 2249]<br>5.8 [5.7 – 6.0]<br>96% [4.0]					
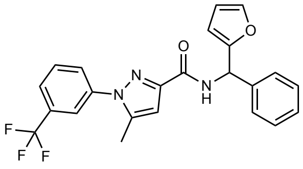 **‘1081**	116 [76.3 – 178]<br>6.9 [6.8 – 7.1]<br>96% [4.0]	--	--	--	--	--
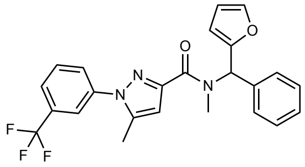 **‘1082**	850 [488 – 1491]<br>6.1 [5.8 – 6.3]<br>94% [5.7]	--	--	--	--	--

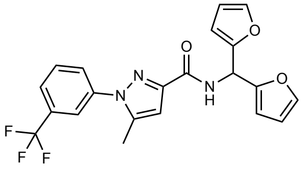 **‘1090**	90.8 [42.7 – 192]<br>7.0 [6.7 – 7.4]<br>99% [7.13]	--	--	--	--	--
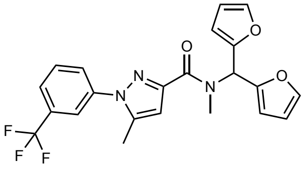 **‘4388**	1360 [998 – 1857]<br>5.9 [5.7 – 6.0]<br>113% [4.4]	--	--	--	--	--
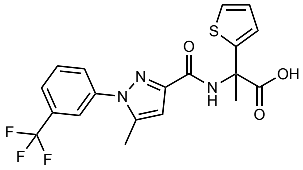 **‘4051**	5328 [3774–7507]<br>5.3 [5.1 – 5.4]<br>103% [3.8]	--	--	--	--	--
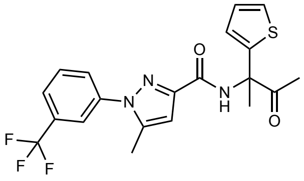 **‘4156**	13.5 [6.3 – 30.1]<br>7.9 [7.5 – 8.2]<br>91% [7.5]					
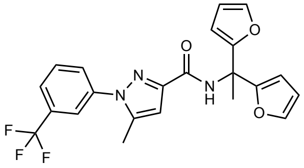 **‘4936**	7.5 [3.9 – 14.3]<br>8.1 [7.9 – 8.4]<br>88% [6.3]					

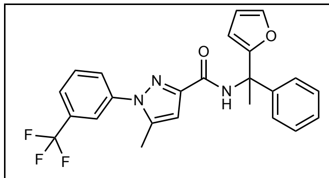<br>**‘5806**	8.0 [3.0 – 21.8]<br>8.1 [7.7 – 8.5]<br>100% [10.5]					
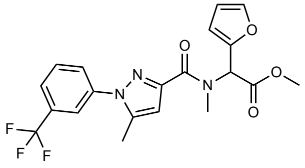<br>**‘6425**	934 [583 – 1501]<br>6.0 [5.8 – 6.2]<br>108% [5.8]	--	--	--	--	--
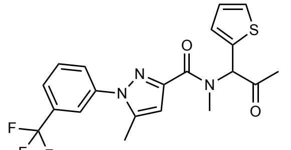<br>**‘6829**	1046 [669 – 1643]<br>6.0 [5.8 – 6.2]<br>106% [5.8]	--	--	--	--	--
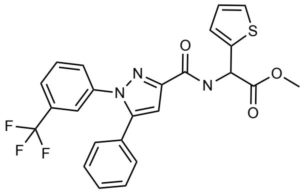<br>**‘8079**	18.5 [13.8 – 25.0]<br>7.7 [7.6 – 7.9]<br>104% [3.0]	--	--	--	--	--
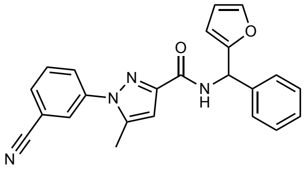<br>**‘12565**	301 [195 – 462]<br>6.5 [6.3 – 6.7]<br>97% [4.0]	--	--	--	--	--

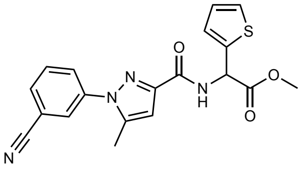<br>**‘31604**	801 [596 –1,076]<br>6.1 [6.0 – 6.2]<br>100% [2.8]	--	--	--	--	--
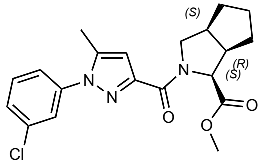<br>**‘10010**	1,196 [952 – 1,505]<br>5.9 [5.8 – 6.0]<br>101% [2.9]	--	--	N.D	> 10,000	--
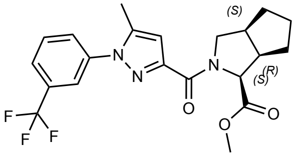<br>**‘6439**	251 [173 – 364]<br>6.6 [6.4 – 6.8]<br>106% [3.9]	--	--	N.D.	> 10,000	--
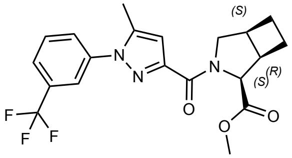<br>**‘6448**	866 [564 – 1,317]<br>6.1 [5.9 – 6.3]<br>103% [4.1]	--	--	--	--	--
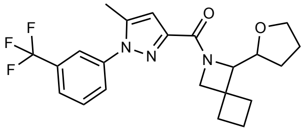<br>**‘3737**	173 [94 – 322]<br>6.76 [6.5 – 7.03]<br>112% [8.8]	--	326 [168 – 1044]<br>6.5 [6.0 – 6.8]<br>99% [91 – 135]	N.D	> 10,000	> 10,000
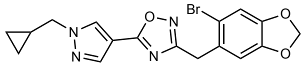<br>**‘5424**	876 [683 – 1123]<br>6.1 [6.0 – 6.2]<br>104% [2.6]	--	--	--	--	--

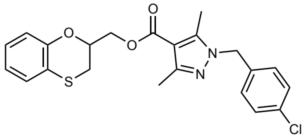 **‘5463**	825 [396 – 1,755]<br>6.1 [5.8 – 6.4]<br>88% [6.6]	--	--	--	--	--
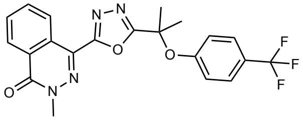 **‘2153**	163 [90 – 287]<br>6.79 [6.5 – 7.0]<br>82.6% [4.5]	--	--	--	--	--
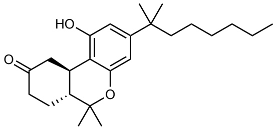 **Nabilone**	2.3 [1.4 – 3.8]<br>8.6 [8.4 – 8.9]<br>81% [3.7]	--	--	--	--	--

N.D. = best fit values were not determined due to compound inactivity or poor data quality
-- Not tested
[a]One-way ANOVA statistical significance of individual comparisons to CP-55,940 after correction with Dunnett’s test of multiple hypotheses. ns = not significant, * p<0.05, ** p<0.01, *** p<0.001, **** p<0.001

Supplementary Table 3. Cryo-EM data collection, model refinement, and validation statistics.

**Data Collection**	**Global Refinement**
Voltage (kV)	300
Magnification	96,000
Total electron dose ($e^-/Å^2$)	56.6
Defocus range (µm)	-0.7 - -2.0
Calibrated pixel size (Å)	0.8521
Micrographs collected	8324
**Data Processing**	
Extracted particles	4,967,593
Particles used for final reconstruction	465,411
Final map resolution (Å, 0.143 FSC)	3.3
Map resolution range (Å)	2.6 - 4.2
Map sharpening B factor ($Å^2$)	175.3
**Model Content**	
Initial models used (PDB code)	6N4B (CB1/G_i/scFv16)
Total number of atoms	8,528
No. of protein residues	1116
No. of ligands	1
**Model Validation**	
CC map vs. model (%)	69.05
RMSD	
Bond lengths (Å) / Bond angles (°)	0.003 / 0.663
Ramachandran plot statistics	
Favored (%)	94.12
Allowed (%)	5.88
Outliers (%)	0.0
Rotamer outliers (%)	0.0
C-beta deviations	0.0
Clash score	7.19

Supplementary Table 4. Functional activities for select analogs versus a variety of transducers and hCB1 in the bioSens-All® platform.

Compound		hCB1 G_{i1}	hCB1 G_{oB}	hCB1 G_z
CP–55,940	EC_{50} [95% CI] (nM)	0.46 [0.4 – 0.5]	0.63 [0.6 – 0.7]	0.28 [0.18 – 0.5]
	pEC_{50} [95% CI]	9.3 [9.3 – 9.4]	9.2 [9.2 – 9.3]	9.6 [9.4 – 9.8]
	E_{max} [95% CI]	100 [98 – 102]	100 [99 – 101]	102 [102 – 104]
‘51486	EC_{50} [95% CI] (nM)	849 [745– 947]	711 [579– 873]	1118 [363– 3447]
	pEC_{50} [95% CI]	6.1 [6.0 – 6.1]	6.2 [6.1 – 6.2]	6.0 [5.5 – 6.4]
	E_{max} [95% CI]	73 [70 – 75]	74 [70 – 75]	130 [996 – 163]
‘60154	EC_{50} [95% CI] (nM)	2.5 [1.9 – 3.4]	18.4 [15.8– 21.5]	17.5 [7.5 – 41]
	pEC_{50} [95% CI]	7.6 [7.5 – 7.7]	7.7 [7.7 – 7.8]	7.8 [7.4 – 8.1]
	E_{max} [95% CI]	92 [90 – 94]	100 [98 – 100]	121 [103 – 140]
‘1081	EC_{50} [95% CI] (nM)	150 [116 – 197]	91.7 [55 – 153]	225 [73 – 694]
	pEC_{50} [95% CI]	6.8 [6.7 – 6.9]	7.0 [6.8 – 7.3]	6.7 [6.2 – 7.1]
	E_{max} [95% CI]	49 [48 – 51]	45 [43 –47]	72 [55 –89]
‘1082	EC_{50} [95% CI] (nM)			
	pEC_{50} [95% CI]	[a]N.D.	N.D.	N.D.
	E_{max} [95% CI]			
‘1087	EC_{50} [95% CI] (nM)			
	pEC_{50} [95% CI]	N.D.	N.D.	N.D.
	E_{max} [95% CI]			
‘1090	EC_{50} [95% CI] (nM)	35.6 [28.5 – 45]	37.3 [30 – 47]	126 [45 – 354]
	pEC_{50} [95% CI]	7.5 [7.3 – 7.5]	7.4 [7.3 – 7.5]	6.9 [6.5 – 7.4]
	E_{max} [95% CI]	81 [80 –82]	90 [88 –92]	115 [92 – 137]
‘4388	EC_{50} [95% CI] (nM)			
	pEC_{50} [95% CI]	N.D.	N.D.	N.D.
	E_{max} [95% CI]			
‘6829	EC_{50} [95% CI] (nM)	4056 [2417 – 9228]	1011 [706 – 1449]	1347 [720 – 2522]
	pEC_{50} [95% CI]	5.4 [5.0 – 5.6]	6.0 [5.8 – 6.2]	5.9 [6.0 – 6.2]
	E_{max} [95% CI]	68 [57 – 77]	49 [45 – 53]	70 [57 –83]
‘4051	EC_{50} [95% CI] (nM)	6523 [5770 – 7511]	7988 [6709 – 9511]	2431 [1069 – 5531]
	pEC_{50} [95% CI]	5.2 [5.1 – 5.2]	5.1 [5.0 – 5.2]	5.6 [5.3 – 6.0]
	E_{max} [95% CI]	108 [104 – 113]	115 [106 – 121]	114 [86 –141]
‘12565	EC_{50} [95% CI] (nM)	104 [79 – 138]	44.8 [27 – 73]	54.6 [22.1 – 135]
	pEC_{50} [95% CI]	7.0 [6.9 – 7.1]	7.4 [7.1 – 7.6]	7.3 [6.9 – 7.7]
	E_{max} [95% CI]	28 [27 – 28]	25 [24 –26]	51 [41 – 61]
‘10010	EC_{50} [95% CI] (nM)	814 [717 – 932]	801 [582– 1102]	1396 [506 – 3853]
	pEC_{50} [95% CI]	6.1 [6.0 – 6.1]	6.1 [6.0 – 6.2]	5.9 [5.4 – 6.3]
	E_{max} [95% CI]	74 [70 – 74]	71 [67 – 75]	144 [113 –175]
‘6439	EC_{50} [95% CI] (nM)	65.5 [56.8 – 755]	60.9 [52.9 – 70]	339 [96 – 1195]
	pEC_{50} [95% CI]	7.2 [7.1 – 7.2]	7.2 [7.2 – 7.3]	6.5 [5.9 – 7.0]
	E_{max} [95% CI]	95 [93 – 96]	100 [99 – 101]	162 [125 –199]
‘6448	EC_{50} [95% CI] (nM)	345 [321 – 372]	310 [267 – 360]	728 [381 – 1393]
	pEC_{50} [95% CI]	6.5 [6.4 – 6.5]	6.5 [6.4 – 6.6]	6.1 [5.9 – 6.4]
	E_{max} [95% CI]	87 [86 – 88]	90 [87 – 91]	151 [130 –172]
‘3737a	EC_{50} [95% CI] (nM)	2804 [2436 – 3285]	6729 [2678 – 16910]	2470 [998 – 6108]
	pEC_{50} [95% CI]	5.6 [5.5 – 5.6]	5.2 [4.8 – 5.6]	5.6 [5.2 – 6.1]
	E_{max} [95% CI]	98 [94 – 102]	118 [94 – 143]	140 [9109 – 172]
‘3737b	EC_{50} [95% CI] (nM)	3026 [2486 – 3822]	19780 [4343 – 90080]	30840 [1133 – 839000]
	pEC_{50} [95% CI]	5.5 [5.4 – 5.6]	4.7 [4.1 – 5.4]	4.5 [3.1– 6.0]
	E_{max} [95% CI]	89 [84 – 94]	152 [94 – 208]	231 [34 – 428]
‘3737c	EC_{50} [95% CI] (nM)			
	pEC_{50} [95% CI]	N.D.	N.D.	N.D.
	E_{max} [95% CI]			
‘3737d	EC_{50} [95% CI] (nM)			
	pEC_{50} [95% CI]	N.D.	N.D.	N.D.
	E_{max} [95% CI]			

**‘7019**	**EC_{50} [95% CI] (nM)**<br>**pEC_{50} [95% CI]**<br>**E_{max} [95% CI]**	N.D.	N.D.	[b]--
**‘5424**	**EC_{50} [95% CI] (nM)**<br>**pEC_{50} [95% CI]**<br>**E_{max} [95% CI]**	N.D.	N,D.	--
**‘7800**	**EC_{50} [95% CI] (nM)**<br>**pEC_{50} [95% CI]**<br>**E_{max} [95% CI]**	6793 [983]<br>5.2 [6.0]<br>34 [24]	597 [398 – 1017]<br>6.2 [5.9 – 6.4]<br>21 [20 – 23]	--
**‘5463**	**EC_{50} [95% CI] (nM)**<br>**pEC_{50} [95% CI]**<br>**E_{max} [95% CI]**	4941 [1763 – 26860000]<br>5.3 [3.4 – 5.8]<br>31 [24 – 88]	7472 [1125]<br>5.1 [6.0]<br>32 [22]	--
**‘0450**	**EC_{50} [95% CI] (nM)**<br>**pEC_{50} [95% CI]**<br>**E_{max} [95% CI]**	56310 [2177]<br>4.3 [5.7]<br>49 [26]	2509 [680 – 11020000]<br>5.6 [3.0 – 6.2]<br>36 [30 – 117]	--
**‘2153**	**EC_{50} [95% CI] (nM)**<br>**pEC_{50} [95% CI]**<br>**E_{max} [95% CI]**	1011 [371 – 6576]<br>6.0 [5.2 – 6.4]<br>89 [78 – 118]	2061 [620 – 25390]<br>5.7 4.6 – 6.2]<br>109 [91 – 162]	--

[a]N.D. = best fit values were not able to be determined due to compound inactivity or poor data quality
[b]-- Not tested

Supplementary Table 5. Detailed functional activities for select analogs and controls versus a variety of transducers and hCB1 in the bioSens-All® platform.

Compound		hCB1 G_{i1}	hCB1 G_{i2}	hCB1 G_{oB}	hCB1 G_z	hCB1 G_{13}	hCB1 G_{15}	hCB1 Barr2 + GRK2
**CP–55,940**	**EC_{50} [95% CI] (nM)**	0.46 [0.4 – 0.5]	0.55 [0.4 – 0.7]	0.63 [0.6 – 0.7]	0.28 [0.18 – 0.5]	2.4 [1.65 – 3.4]	0.26 [0.22 – 2.9]	3.1 [1.97 – 4.8]
	**pEC_{50} [95% CI]**	9.34 [9.3 – 9.4]	9.26 [9.1 – 9.4]	9.20 [9.2 – 9.3]	9.55 [9.4 – 9.8]	8.62 [8.5 – 8.8]	9.59 [9.5 – 9.7]	8.51 [8.3 – 8.7]
	**E_{max} [95% CI]**	100 [98 – 102]	100 [96 – 103]	100 [98 – 101]	101 [96 – 107]	100 [95 – 105]	100 [98 – 102]	89 [82 – 98]
**‘4042**	**EC_{50} [95% CI] (nM)**	0.48 [0.4 – 0.6]	0.56 [0.4 – 0.9]	0.64 [0.5 – 0.8]	0.43 [0.3 – 0.6]	2.1 [0.5 – 9.6]	0.37 [0.28 – 0.49]	3.6 [2.1 – 6.8]
	**pEC_{50} [95% CI]**	9.32 [9.2 – 9.4]	9.25 [9.0 – 9.5]	9.20 [9.1 – 9.3]	9.37 [9.3 – 9.4]	8.69 [8.0 – 9.3]	9.43 [9.3 – 9.6]	8.44 [8.2 – 8.7]
	**E_{max} [95% CI]**	102 [100 – 104]	101 [95 – 106]	103 [99 – 106]	97 [93 – 102]	64 [54 – 84]	105 [102 – 108]	72 [71 – 74]
**‘1350**	**EC_{50} [95% CI] (nM)**	0.23 [0.18 – 0.3]	0.28 [0.19 – 0.4]	0.29 [0.24 – 0.34]	0.35 [0.22 – 0.53]	0.83 [0.23 – 2.7]	0.22 [0.18 – 0.27]	2.2 [1.1 – 4.4]
	**pEC_{50} [95% CI]**	9.63 [9.5 – 9.7]	9.54 [9.4 – 9.7]	9.54 [9.5 – 9.6]	9.46 [9.3 – 9.7]	9.08 [8.6 – 9.6]	9.66 [9.6 – 9.8]	8.66 [8.4 – 9.0]
	**E_{max} [95% CI]**	92 [90 – 95]	95 [90 – 99]	98 [94 – 98]	94 [88 – 100]	56 [48 – 67]	99 [96 – 101]	63 [60 – 72]
**‘8690**	**EC_{50} [95% CI] (nM)**	19.1 [14.4 – 25.8]	[b]--	18 [14 – 23]	33 [15 – 117]	--	--	[a]N.D.
	**pEC_{50} [95% CI]**	7.72 [7.6 – 7.8]		7.8 [7.7 – 7.9]	7.5 [6.9 – 7.8]			
	**E_{max} [95% CI]**	82 [77 – 85]		83 [80 – 87]	98 [86 – 121]			
**2-AG**	**EC_{50} [95% CI] (nM)**	224 [147 – 353]	62 [42 – 918]	93 [65 – 135]	493 [15]	4470 [1618 – 398200]	32 [25 – 40]	1025 [774 –1446]
	**pEC_{50} [95% CI]**	6.65 [6.5 – 6.8]	7.2 [7.0 – 7.4]	7.0 [6.9 – 7.2]	6.3 [7.8]	5.4 [5.1 – 5.8]	7.5 [7.4 – 7.6]	6.0 [5.8 – 6.1]
	**E_{max} [95% CI]**	122 [113 – 133]	112 [106 – 119]	114 [108 – 121]	205 [112]	183 [133 – 607]	112 [108 – 116]	200 [184 – 220]

[a]N.D. = best fit values were not able to be determined due to compound inactivity or poor data quality

[b]-- Not tested

Supplementary Table 6. Relative efficacy calculations for ‘4042 and ‘1350 versus CP-55,940.

Target	Sensor	Compound	Mean log (E_{max}/EC_{50})	SEM log (E_{max}/EC_{50})	Mean Δlog (E_{max}/EC_{50})	SEM Δlog (E_{max}/EC_{50})	t-test to CP-55,940[a]	RE[b]
hCB1	G_{i1}	**CP-55,940**	9.34	0.07	0.00	0.09		1.00
		**'4042**	9.33	0.14	-0.01	0.15	ns	0.97
		**'1350**	9.59	0.06	0.30	0.09	****	1.98
	G_{i2}	**CP-55,940**	9.24	0.13	0.00	0.18		1.00
		**'4042**	9.25	0.03	0.01	0.13	ns	1.01
		**'1350**	9.52	0.03	0.28	0.13	**	1.89
	G_{oB}	**CP-55,940**	9.19	0.07	0.00	0.10		1.00
		**'4042**	9.21	0.09	0.02	0.11	ns	1.04
		**'1350**	9.09	0.44	0.35	0.44	****	2.24
	G_z	**CP-55,940**	9.53	0.23	0.00	0.33		1.00
		**'4042**	9.32	0.19	-0.21	0.30	ns	0.62
		**'1350**	9.42	0.22	-0.11	0.32	ns	0.77
	G_{13}	**CP-55,940**	8.63	0.23	0.00	0.32		1.00
		**'4042**	8.59	0.25	-0.04	0.33	ns	0.91
		**'1350**	8.85	0.05	0.22	0.23	*	1.64
	G_{15}	**CP-55,940**	9.59	0.02	0.00	0.02		1.00
		**'4042**	9.46	0.12	-0.13	0.12	ns	0.74
		**'1350**	9.65	0.06	0.06	0.06	ns	1.14
	$ß_{arr2}$+ GRK2	**CP-55,940**	8.28	0.25	0.00	0.35		1.00
		**'4042**	8.19	0.03	-0.09	0.03	*	0.81
		**'1350**	8.34	0.08	0.06	0.08	ns	1.16
hCB2	G_{i1}	**CP-55,940**	8.86	0.12	0.00	0.17		1.00
		**'4042**	8.49	0.03	-0.20	0.13	ns	0.63
		**'1350**	8.32	0.10	-0.54	0.16	*	0.29
	G_{i2}	**CP-55,940**	8.97	0.00	0.00	0.00		1.00
		**'4042**	8.74	0.00	-0.23	0.00	[c]--	0.59
		**'1350**	8.57	0.00	-0.40	0.00	--	0.40
	G_{oB}	**CP-55,940**	8.76	0.09	0.00	0.13		1.00
		**'4042**	8.43	0.19	-0.33	0.21	n.s.	0.47
		**'1350**	8.36	0.01	-0.40	0.09	***	0.40
	G_z	**CP-55,940**	8.90	0.39	0.00	0.55		1.00
		**'4042**	8.48	0.51	-0.42	0.64	n.s.	0.38
		**'1350**	8.24	0.23	-0.65	0.45	n.s.	0.22
	$ß_{arr2}$+ GRK2	**CP-55,940**	7.83	0.13	0.00	0.18		1.00
		**'4042**	7.74	0.00	-0.09	0.13	***	0.82
		**'1350**	7.79	0.09	-0.04	0.16	n.s.	0.92

[a]Statistical significance of all comparisons of compound activities (Mean Δlog (E_{max}/EC_{50})) to CP-55,940 control by unpaired t-test. ns = not significant, * p<0.05, ** p<0.01, *** p<0.001, **** p<0.0001

[b]RE, relative efficacy = $10^{\Delta \log (Emax/EC50)}$

[c]--, not determined

Supplementary Table 7. Binding affinities and functional activities for select active analogs at CB2.

Compound	**rCB2 binding**<br>EC_{50} [95% CI] (nM)<br>pEC_{50} [95% CI]<br>E_{max} [SEM]	**hCB2<br>Cerep cAMP**<br>EC_{50} [95% CI] (nM)<br>pEC_{50} [95% CI]<br>E_{max} [95% CI]	**hCB2<br>BRET2 + GoA**<br>EC_{50} [95% CI] (nM)<br>pEC_{50} [95% CI]<br>E_{max} [95% CI]	**hCB2<br>Tango<br>ß-arrestin<br>recruitment**<br>EC_{50} [95% CI] (nM)<br>pEC_{50} [95% CI]<br>E_{max} [95% CI]
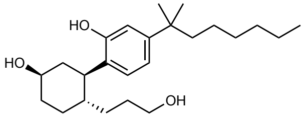<br>**CP–55,940**	--	0.082	13.1 [9.4 – 18.5]<br>7.9 [7.7 – 8.0]<br>99% [94 – 105]	22.9 [21.3 – 24.8]<br>7.6 [7.60 – 7.67]<br>100% [98 – 102]
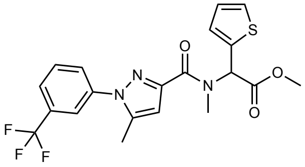<br>**‘4042**	2.18 [1.7 – 2.8]<br>8.7 [8.6 – 8.8]<br>103% [8.8]	0.011 [0.002 – 0.02]<br>10.95 [10.7 – 11.6]<br>72% [77 – 84]	7.9 [1.6 – 40.6]<br>8.1 [7.4 – 8.8]<br>29% [22 – 36]	33.9 [21.7 – 61.5]<br>7.5 [7.2 – 7.7]<br>28% [26 – 32]
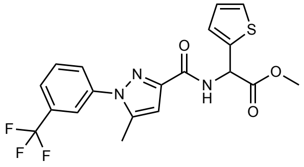<br>**‘60154**	[b]--	--	594 [125 – 2813]<br>6.2 [5.6 – 6.9]<br>31% [20 – 42]	3341 [2707 – 4371]<br>5.5 [5.4 – 5.6]<br>21% [19 – 23]
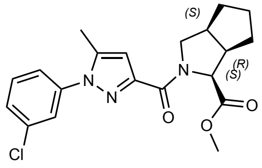<br>**10010**	--	--	[a]N.D.	556 [506 – 611]<br>6.3 [6.2 – 6.3]<br>18% [17 – 19]

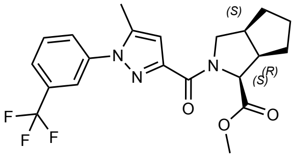 **‘6439**	--	--	97.1 [12.6 – 749]<br>7.0 [6.13 – 67.9]<br>81% [34 – 128]	338 [282– 415]<br>6.5 [6.4 – 6.6]<br>14% [13 – 15]
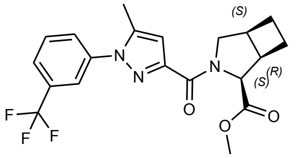 **‘6448**	--	--	--	--
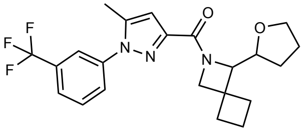 **‘3737**	--	4.3 [.0004 – 22.6]<br>8.4 [7.7 – 12.5]<br>45% [42 – 80%]	N.D.	1500 [1274 – 1805]<br>5.8 [5.7 – 5.9]<br>9% [8 – 9]

[a]N.D. = best fit values were not determined due to compound inactivity or poor data quality
[b]-- Not tested

Supplementary Table 8. Functional activities for select analogs and controls versus a variety of transducers and hCB2 in the bioSensAll platform.

Compound		hCB2 G_{i1}	hCB2 G_{i2}	hCB2 G_{oB}	hCB2 G_z	hCB2 Barr2 + GRK2
**CP–55,940**	**EC_{50} [95% CI] (nM)**	1.4 [1.1 – 1.7]	1.1 [0.9 – 1.3]	1.7 [1.5 – 2.1]	1.05 [0.6 – 1.9]	14.3 [11.6 – 17.9]
	**pEC_{50} [95% CI]**	8.87 [8.7 – 8.9]	8.97 [8.9 – 9.1]	8.76 [8.7 – 8.8]	8.98 [8.7 – 9.2]	7.84 [7.8 – 7.9]
	**E_{max} [95% CI]**	100 [97 – 103]	100 [97 – 104]	100 [98 – 103]	98 [89 – 109]	100 [95 – 104]
**‘4042**	**EC_{50} [95% CI] (nM)**	2.7 [2.0 – 3.4]	1.3 [0.9 – 1.7]	2.5 [1.9 – 3.3]	1.36 [0.5 – 3.1]	5.5 [3.5 – 8.7]
	**pEC_{50} [95% CI]**	8.58 [8.5 – 8.7]	8.90 [8.8 – 9.0]	8.60 [8.5 – 8.7]	8.87 [8.5 – 9.3]	8.26 [8.1 – 8.5]
	**E_{max} [95% CI]**	82 [79 – 85]	70 [67 – 73]	61 [59 – 63]	56 [47 – 68]	33 [33 – 33]
**‘1350**	**EC_{50} [95% CI] (nM)**	3.55 [2.98 – 4.2]	1.6 [1.4 – 1.9]	2.6 [2.15 – 3.1]	2.6 [1.6 – 3.9]	4.2 [2.8 – 6.3]
	**pEC_{50} [95% CI]**	8.45 [8.4 – 8.5]	8.79 [8.7 – 8.9]	8.58 [8.5 – 8.7]	8.58 [8.4 – 8.8]	8.38 [8.2 – 8.6]
	**E_{max} [95% CI]**	74 [72 – 77]	61 [59 –63]	59 [57 – 61]	52 [46 – 60]	30 [28 –33]
**2-AG**	**EC_{50} [95% CI] (nM)**	217 [186 – 256]	96 [70 – 129]	394 [349 – 447]	2123 [931 – 87070]	1854 [1110–4061]
	**pEC_{50} [95% CI]**	6.66 [6.6 – 6.7]	7.0 [6.9 – 7.2]	6.4 [6.3 – 6.5]	5.7 [4.1 – 6.0]	5.7 [5.4 – 6.0]
	**E_{max} [95% CI]**	105 [102 – 109]	78 [74 – 84]	101 [98 – 104]	96 [76 – 233]	118 [104 – 140]
