# Supplementary material for: Large library docking for cannabinoid-1 receptor agonists with reduced side effects
Source: bioRxiv. 2024 Jan 23:2023.02.27.530254. Preprint. [Version 2] doi: 10.1101/2023.02.27.530254 (PMC10849508; doi:10.1101/2023.02.27.530254)
Supplement: Supplement 3 [file NIHPP2023.02.27.530254v2-supplement-3.pdf]

bioRxiv preprint doi: https://doi.org/10.1101/2023.02.27.530254; this version posted January 23, 2024. The copyright holder for this preprint (which was not certified by peer review) is the author/funder, who has granted bioRxiv a license to display the preprint in perpetuity. It is made available under aCC-BY 4.0 International license.

Supplemental Data Figures

A

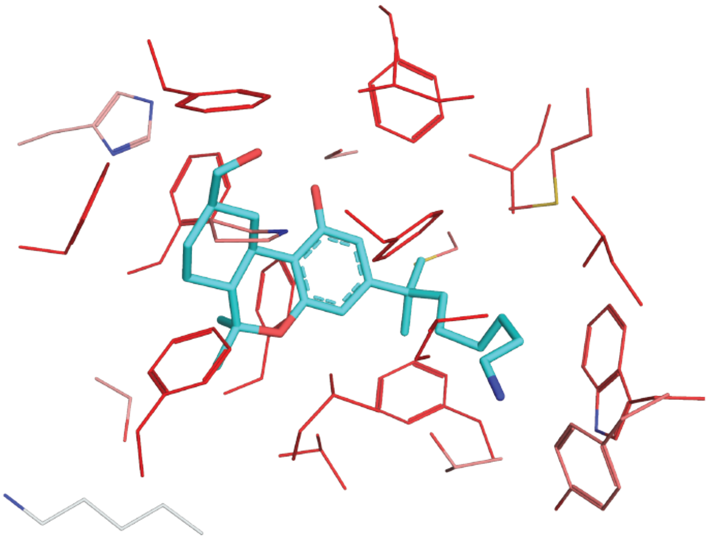

B

Residue Number	Residue Type	Polarity	Kyte & Doolittle Scale [-4.5-4.5]	Eisenberg Scale [-2.5-1.38]
108	PHE	NP	2.80	1.19
170	PHE	NP	2.80	1.19
173	SER	P	-0.80	-0.18
174	PHE	NP	2.80	1.19
177	PHE	NP	2.80	1.19
178	HIS	C/P	-3.20	-0.4
189	PHE	NP	2.80	1.19
192	LYS	C	-3.90	-1.5
193	LEU	NP	3.80	1.06
196	VAL	NP	4.20	1.08
197	THR	P	-0.70	-0.05
200	PHE	NP	2.80	1.19
267	ILE	NP	4.50	1.38
268	PHE	NP	2.80	1.19
269	PRO	NP	-1.60	0.12
271	ILE	NP	4.50	1.38
275	TYR	P	-1.30	0.26
276	LEU	NP	3.80	1.06
279	TRP	NP	-0.90	0.81
359	LEU	NP	3.80	1.06
363	MET	NP	1.90	0.64
379	PHE	NP	2.80	1.19
383	SER	P	-0.80	-0.18
386	CYS	NP	2.50	0.29
		Average	1.59	0.68

Figure S1. Hydrophobicity calculations for the hCB1R orthosteric pocket based on PDB: 5XR8. Residues within 5 Å of AM841 are considered. **A.** Depiction of the hCB1 orthosteric pocket, colored by the Eisenberg Scale, where darker red colors indicate more hydrophobic residues and lighter red or gray colors indicate less hydrophobic residues. **B.** A table of the residues within 5 Å of AM841, with their polarity class, and two hydrophobicity scores indicated.

bioRxiv preprint doi: https://doi.org/10.1101/2023.02.27.530254; this version posted January 23, 2024. The copyright holder for this preprint (which was not certified by peer review) is the author/funder, who has granted bioRxiv a license to display the preprint in perpetuity. It is made available under aCC-BY 4.0 International license.

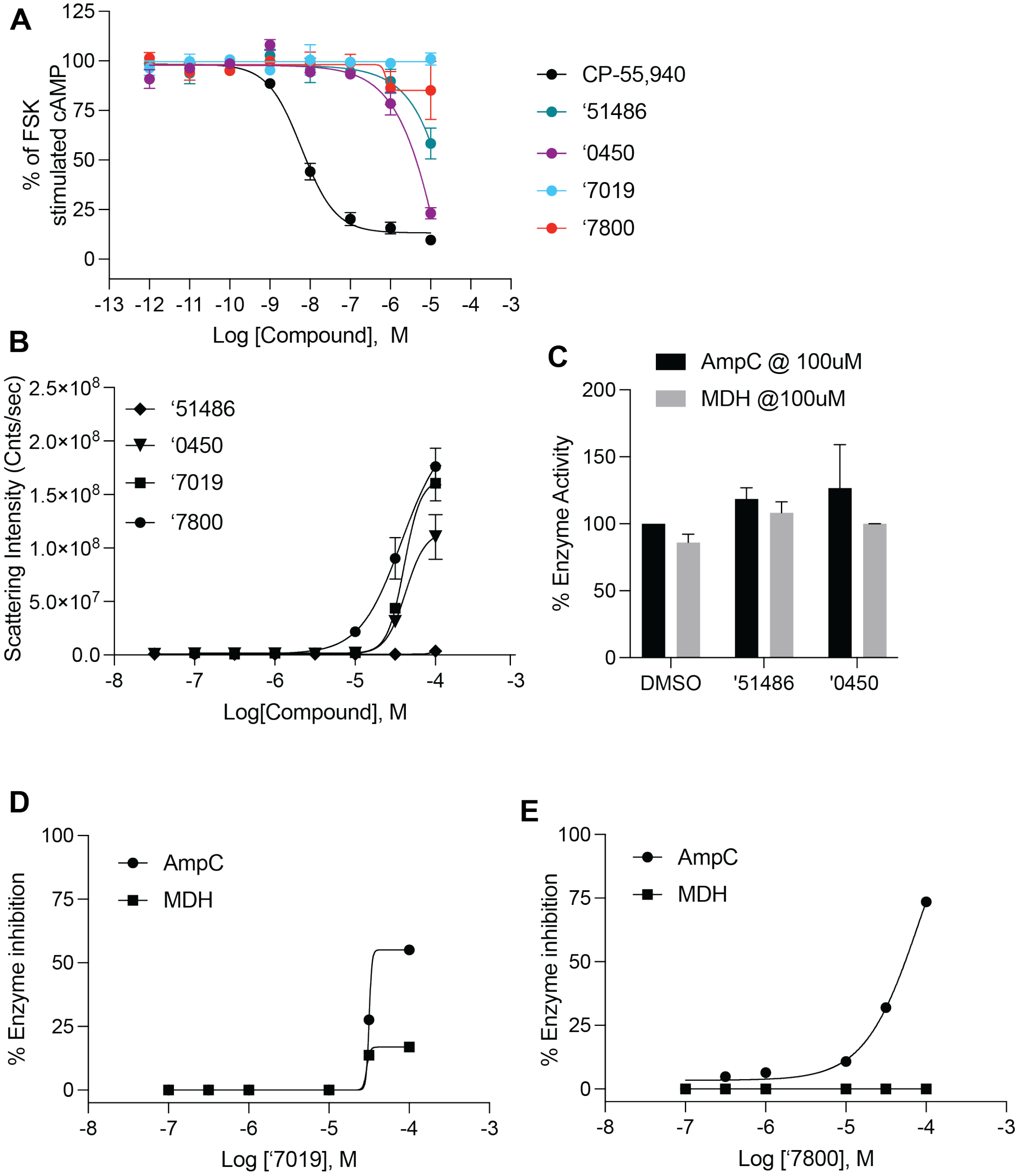

Figure S2. Functional measurements for a subset of screening hits. **A.** Functional cAMP inhibition at hCB1R by the four most potent docking hits. **B.** Scattering intensity in dynamic light scattering experiments of colloidal aggregation. **C.** Inhibition of the off-target enzymes MDH and AmpC Beta-lactamase at 100 uM. **D.** and **E.** Single-point inhibition of the off-target enzymes MDH and AmpC Beta-lactamase by **'7019 (D.)** and **'7800 (E.).** All data represent mean ± SEM of three independent experiments in triplicate except **B.** which represents one independent experiment in triplicate.

bioRxiv preprint doi: https://doi.org/10.1101/2023.02.27.530254; this version posted January 23, 2024. The copyright holder for this preprint (which was not certified by peer review) is the author/funder, who has granted bioRxiv a license to display the preprint in perpetuity. It is made available under aCC-BY 4.0 International license.

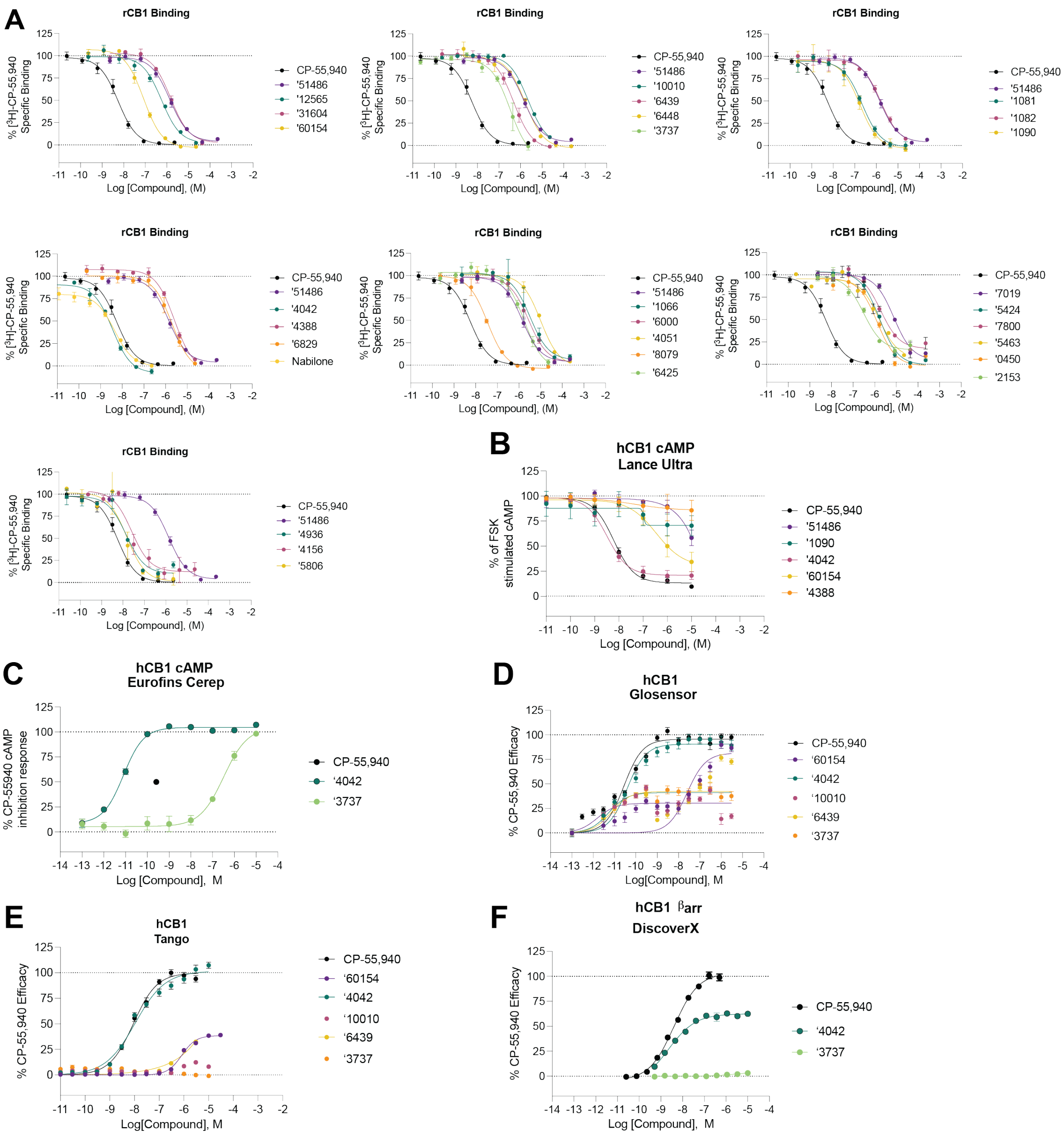

Figure S3. hCB1 binding and functional data for analogs. **A.** Competition binding data for primary hits and a subset of their analogs at hCB1. **B-D.** Functional cAMP inhibition for a subset of analogs at hCB1 across three separate assays. **E-F.** Functional ß$_{arr}$ recruitment for a subset of analogs. All data represent mean ± SEM of at least 2 independent experiments in triplicate except **C.** and **F.** which represent one independent experiment in triplicate. Best fit values can be found in **Supplementary Table 2**.

bioRxiv preprint doi: https://doi.org/10.1101/2023.02.27.530254; this version posted January 23, 2024. The copyright holder for this preprint (which was not certified by peer review) is the author/funder, who has granted bioRxiv a license to display the preprint in perpetuity. It is made available under aCC-BY 4.0 International license.

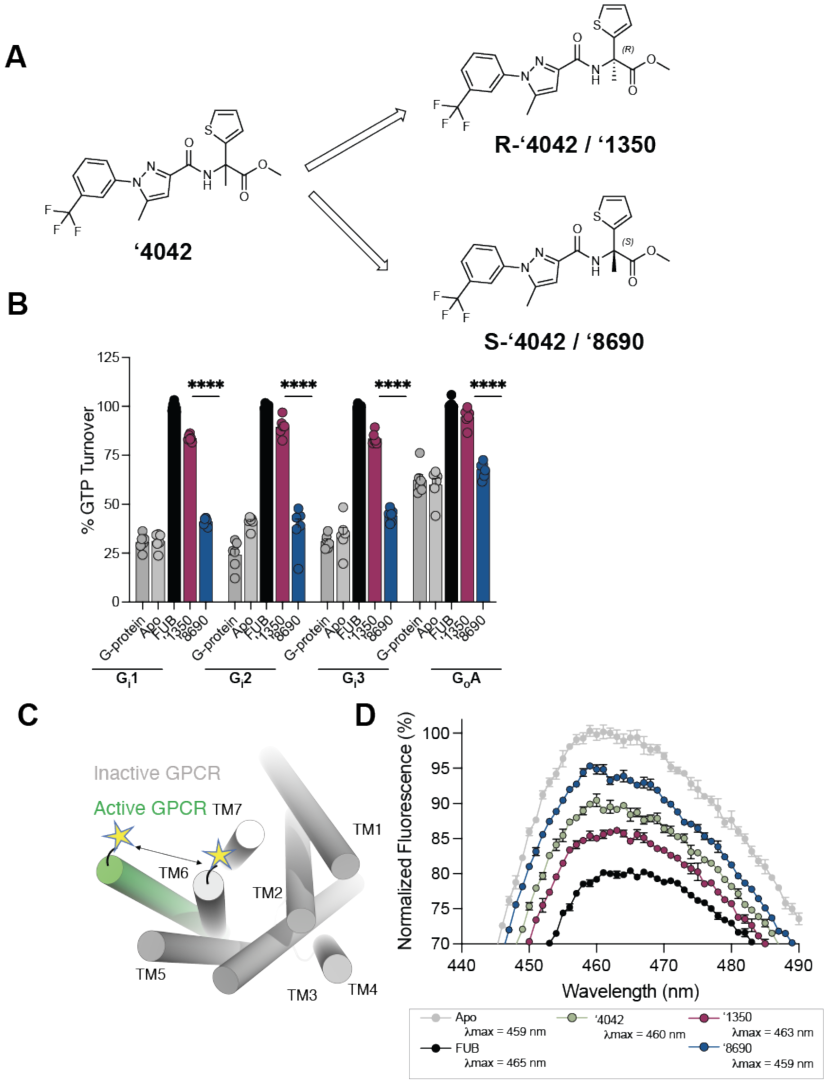

Figure S4. Additional pharmacological characterization of **'4042** and its enantiomers. **A.** Chiral column purification led to the separation of two independent enantiomers, **'1350** and **'8690**. '**1350** was determined to be ***R*-'4042** from the Cryo-EM structure. **B.** GTPase Glo assay characterizing GTP turnover of G-proteins $G_{i1\text{-}3/o}$. **C.** Schematic of the environmentally sensitive fluorophore Monobromobimane (Bimane) which when site-specifically labeled (e.g. on TM6) acts as a conformational reporter. **D**. Compared to the apo (grey), the spectrum of full agonist MDMB-fubinaca (Fub)-bound CB1 (black) shows a decrease in intensity and a blue-shift in λ_{max} (Apo 459 nm to Fub 465 nm). The bimane spectrum of **'8690** (λ_{max} 459 nm, blue) is more similar to apo and the spectrum of **'1350** (λ_{max} 463 nm, magenta) is closer to that of Fub. The spectrum of the racemate, **'4042** (green) is between **'1350** (***R*-'4042**) and **'8690** (***S*-'4042**). All data represent mean ± SEM of three independent experiments in triplicate.

bioRxiv preprint doi: https://doi.org/10.1101/2023.02.27.530254; this version posted January 23, 2024. The copyright holder for this preprint (which was not certified by peer review) is the author/funder, who has granted bioRxiv a license to display the preprint in perpetuity. It is made available under aCC-BY 4.0 International license.

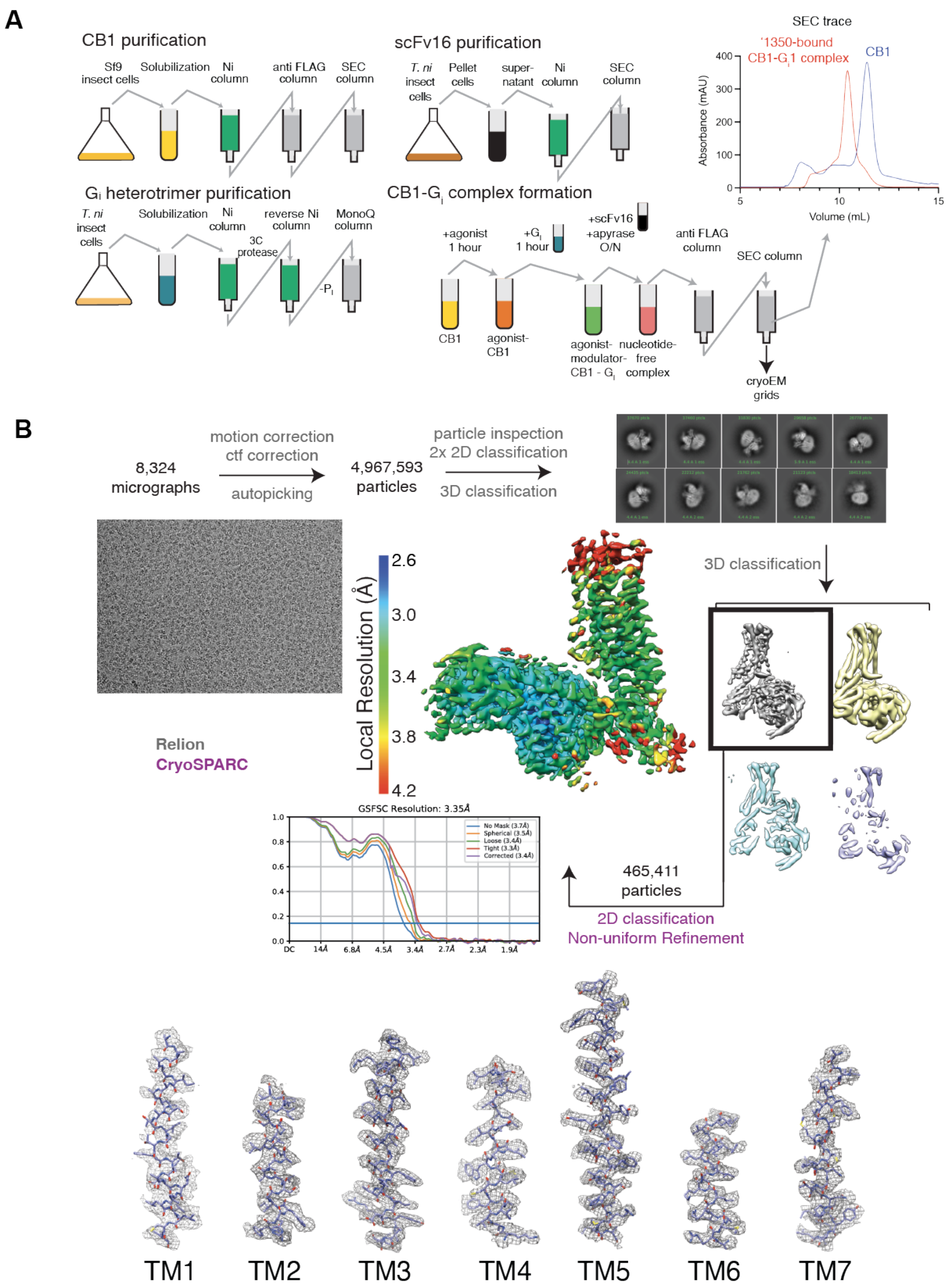

Figure S5. Cryo-EM sample preparation and data processing. **A.** Purification of hCB1, scFv16, the G_i heterotrimer, and complex formation protocols. **B.** Cryo-EM data processing flow chart of CB1, including particle selection, classifications, and density map reconstruction. Details can be found in **Supplementary Table 3**.

bioRxiv preprint doi: https://doi.org/10.1101/2023.02.27.530254; this version posted January 23, 2024. The copyright holder for this preprint (which was not certified by peer review) is the author/funder, who has granted bioRxiv a license to display the preprint in perpetuity. It is made available under aCC-BY 4.0 International license.

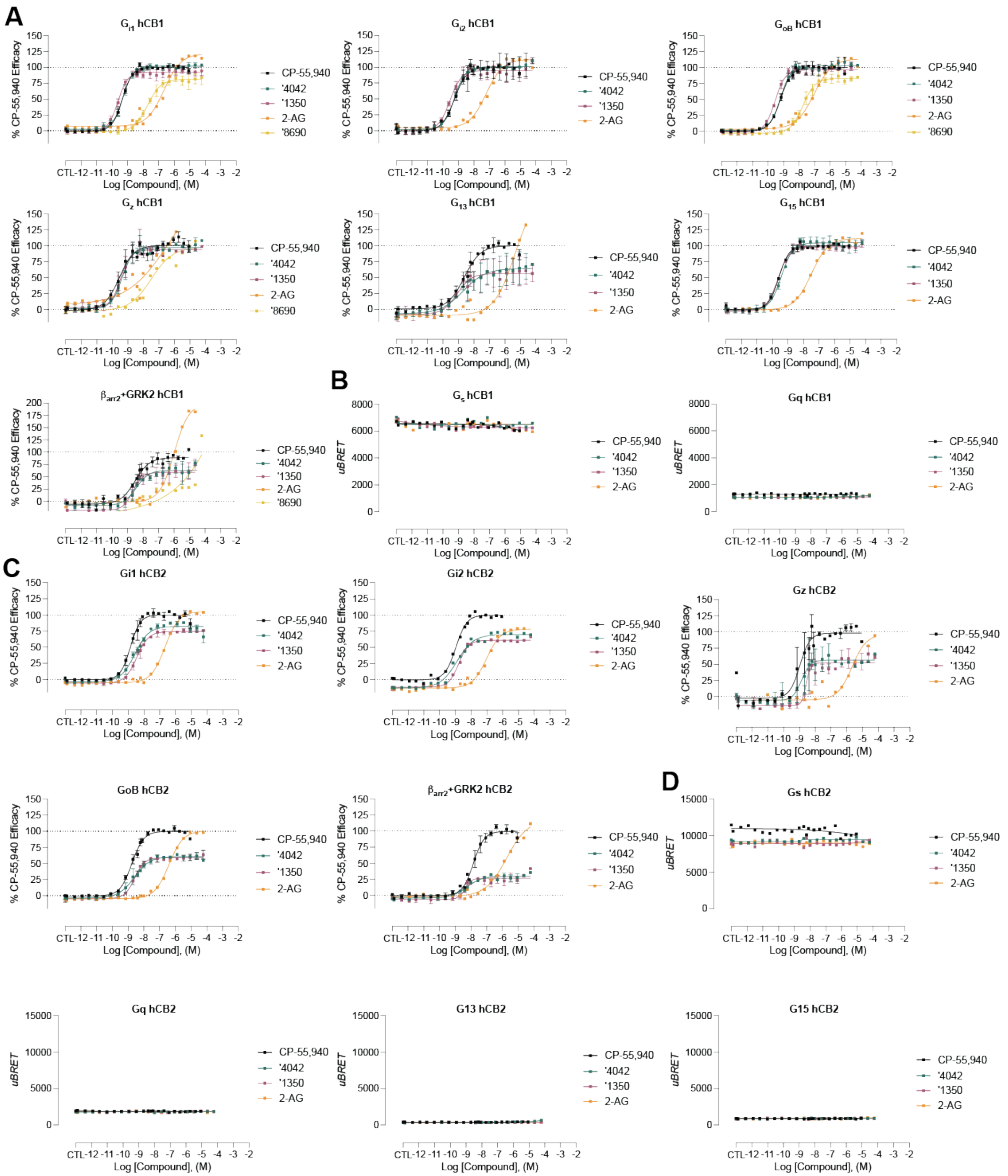

Figure S6. hCB1/2 functional data for select analogs in the bioSens-All® platform. **A.** Normalized activity for select analogs versus a panel of sensors in hCB1-expressing cells. **B.** Raw BRET activity for select ana- logs versus G_s and G_q in hCB1-expressing cells. **C.** Normalized activity for select analogs versus a panel of sensors in hCB2-expressing cells. **D.** Raw BRET activity for select analogs versus G_s, G_q, G_{12}, and G_{15} in hCB2-expressing cells. Best fit values can be found in **Supplementary Tables 5 & 8**.

bioRxiv preprint doi: https://doi.org/10.1101/2023.02.27.530254; this version posted January 23, 2024. The copyright holder for this preprint (which was not certified by peer review) is the author/funder, who has granted bioRxiv a license to display the preprint in perpetuity. It is made available under aCC-BY 4.0 International license.

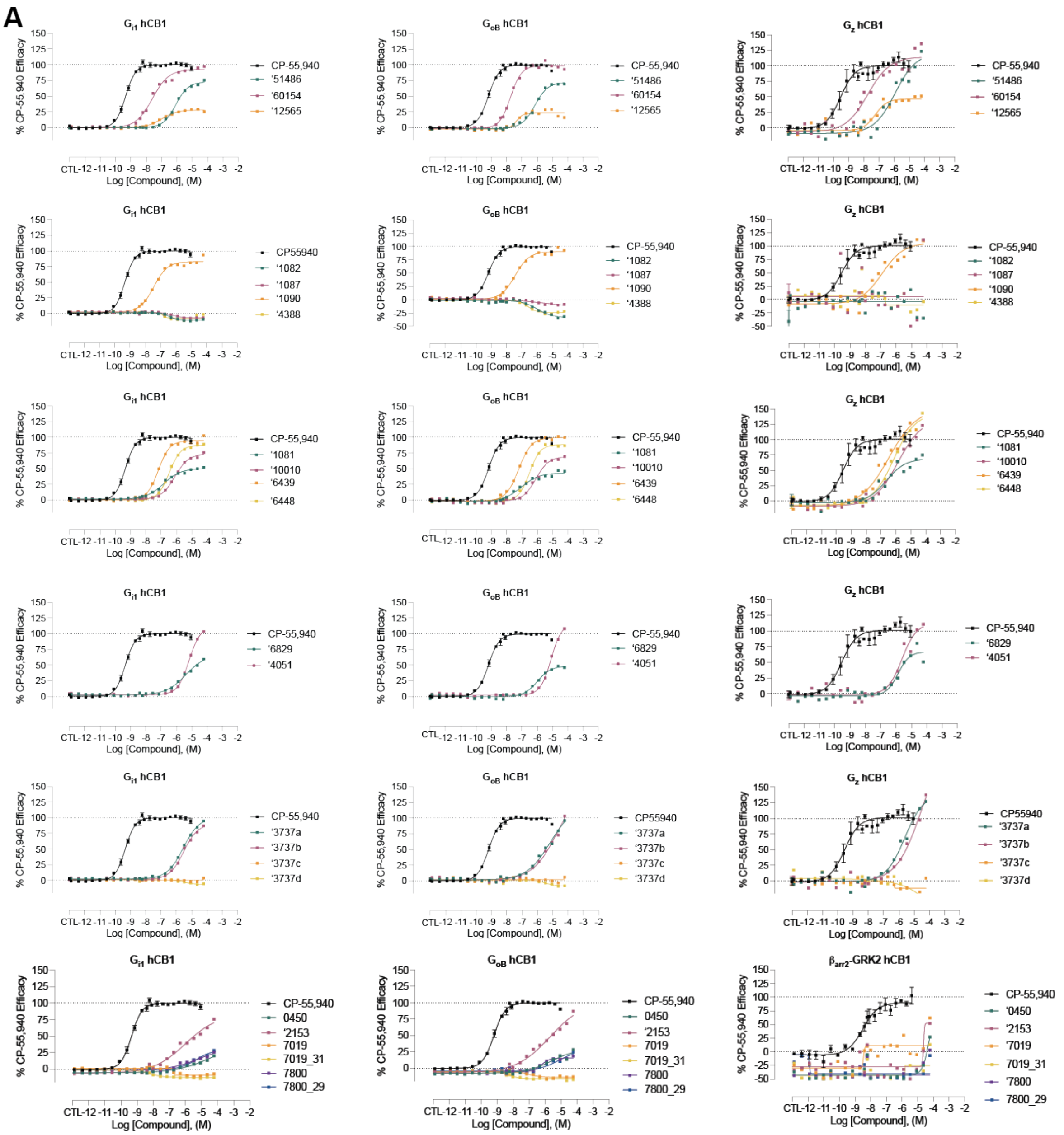

Figure S7. hCB1 functional data for select analogs in the bioSens-All® platform. **A.** Normalized activity for select analogs versus a panel of sensors in hCB1-expressing cells. Best fit values can be found in **Supplementary Table 4**.

bioRxiv preprint doi: https://doi.org/10.1101/2023.02.27.530254; this version posted January 23, 2024. The copyright holder for this preprint (which was not certified by peer review) is the author/funder, who has granted bioRxiv a license to display the preprint in perpetuity. It is made available under aCC-BY 4.0 International license.

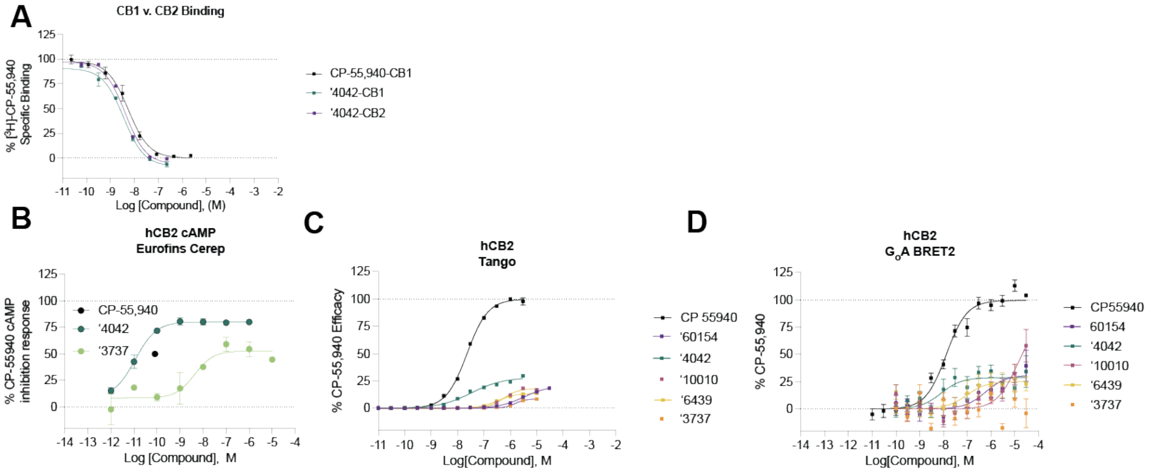

Figure S8. CB2R binding and functional data for select analogs. **A.** Competition binding data shows that **'4042** is modestly more potent at CB1R than CB2R (rCB1 pKi = 8.7 (95% CI 8.60 – 8.86), hCB2 pKi = 8.6 (95% CI 8.55 – 8.77); $t(4) = 6.5$, $p = 0.003$). **B-D.** Functional cAMP inhibition for a subset of analogs at hCB2 across three separate assays. All data represent mean ± SEM of three independent experiments in triplicate except **B.** which represents one independent experiment in triplicate. Best fit values can be found in **Supplementary Table 7**.

bioRxiv preprint doi: https://doi.org/10.1101/2023.02.27.530254; this version posted January 23, 2024. The copyright holder for this preprint (which was not certified by peer review) is the author/funder, who has granted bioRxiv a license to display the preprint in perpetuity. It is made available under aCC-BY 4.0 International license.

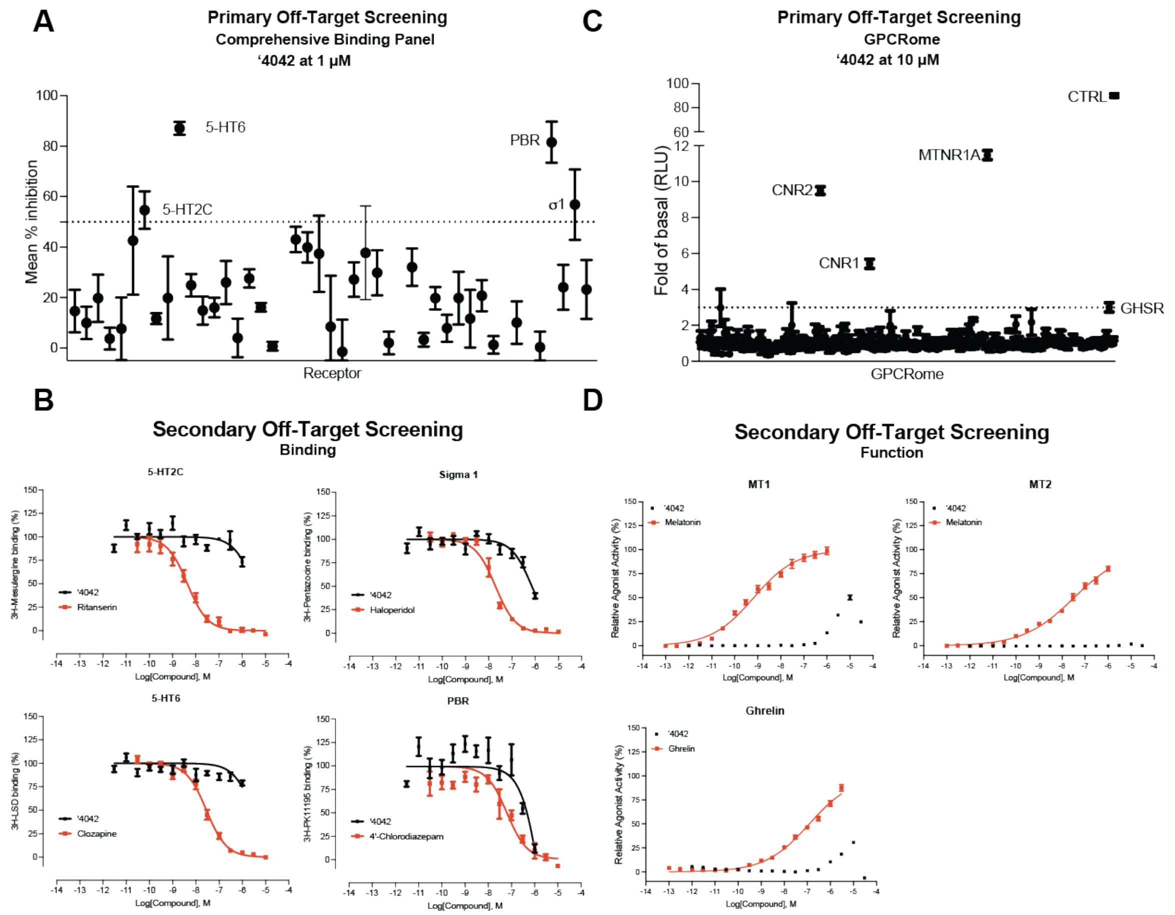

Figure S9. Off-target profiling of **'4042**. **A.** Comprehensive binding data against a panel of 45 common GPCR and non-GPCR drug targets. **B.** Follow-up dose response binding experiments for targets with > 50% inhibition in the single-point experiments. **C.** TANGO screens against a panel of 320 GPCRs for **'4042**. **D.** Follow-up dose response functional experiments for targets with > 3-fold activation in the single-point experiments. Data in **A.**, **C.,** and **D.** represent mean ± SEM of 3 independent experiments in triplicate. Data in **B.** represent mean ± SEM of 2 independent experiments in triplicate except 5-HT6 which is 3 independent experiments in triplicate.

bioRxiv preprint doi: https://doi.org/10.1101/2023.02.27.530254; this version posted January 23, 2024. The copyright holder for this preprint (which was not certified by peer review) is the author/funder, who has granted bioRxiv a license to display the preprint in perpetuity. It is made available under aCC-BY 4.0 International license.

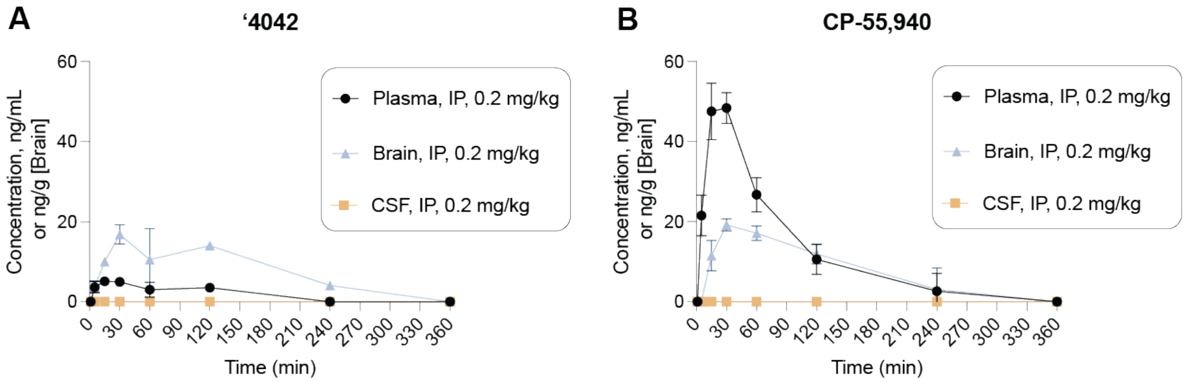

Sample	Administration	Dose, mg/kg	'4042 Pharmacokinetic Parameters					
			Tmax, Min	Cmax, ng/ml (g)	$AUC_{0\to t}$ (AUClast) ng*min/ml (g)	$AUC_{0\to\infty}$ (AUCINF_obs), ng*min/ml (g)	$T_{1/2}$ (HL_Lambda_z), min	K_{el} (Lambda_z), min^{-1}
Plasma	IP	0.2	15.0	5.14	446	1350	178	0.00388
Brain	IP	0.2	30.0	16.8	2510	3180	114	0.00609
CSF	IP	0.2	ND	BQL	BQL	ND	ND	ND

Sample	Administration	Dose, mg/kg	CP-55,940 Pharmacokinetic Parameters					
			Tmax, Min	Cmax, ng/ml (g)	$AUC_{0\to t}$ (AUClast) ng*min/ml (g)	$AUC_{0\to\infty}$ (AUCINF_obs), ng*min/ml (g)	$T_{1/2}$ (HL_Lambda_z), min	K_{el} (Lambda_z), min^{-1}
Plasma	IP	0.2	30.0	48.4	3360	3990	41.5	0.0167
Brain			30.0	19.2	1700	3880	127	0.098
CSF			ND	BQL	BQL	ND	ND	ND

Figure S10. Pharmacokinetic profiles of **'4042** compared to CP-55.940**.** Pharmacokinetic profile of **'4042** (**A**.) and CP-55,940 (**B**.) after a single 0.2 mg/kg dose in brain, CSF, and plasma compartments. Data represent mean ± SEM of 3 animals per timepoint.

bioRxiv preprint doi: https://doi.org/10.1101/2023.02.27.530254; this version posted January 23, 2024. The copyright holder for this preprint (which was not certified by peer review) is the author/funder, who has granted bioRxiv a license to display the preprint in perpetuity. It is made available under aCC-BY 4.0 International license.

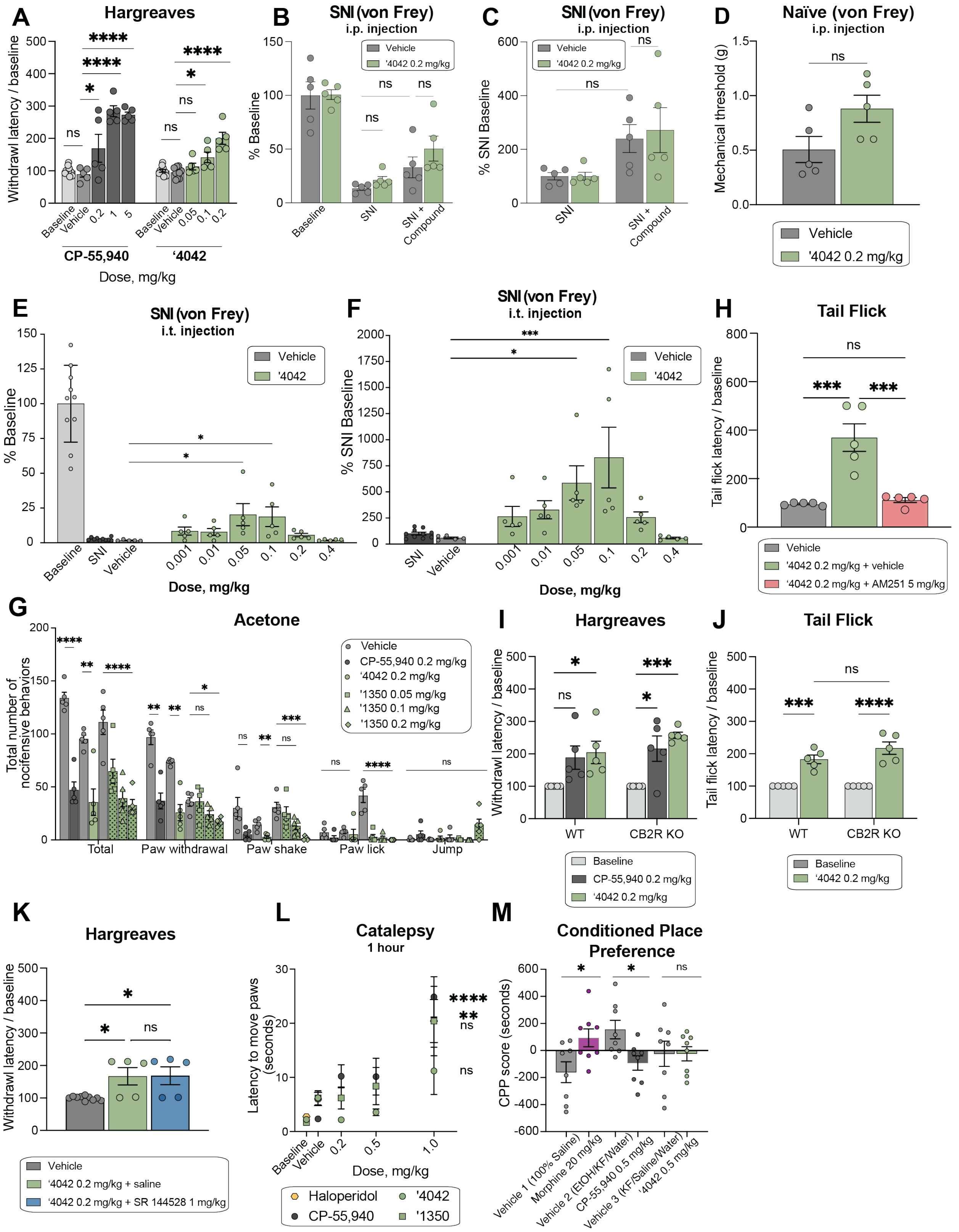

bioRxiv preprint doi: https://doi.org/10.1101/2023.02.27.530254; this version posted January 23, 2024. The copyright holder for this preprint (which was not certified by peer review) is the author/funder, who has granted bioRxiv a license to display the preprint in perpetuity. It is made available under aCC-BY 4.0 International license.

Figure S11. Additional analgesic and side-effect profiles of **‘4042** and **‘1350. A.** Dose-response activity in the Hargreaves assay for **‘4042** (n = 5; one-way ANOVA, $F(3, 21) = 16.3$, $P < 0.0001$) and CP-55,940 (n = 5; one-way ANOVA, $F(4, 25) = 26.2$, $P < 0.0001$). Asterisks define individual group differences to respective vehicle control using Dunnett’s multiple comparisons post-hoc test correction. **B.** Effect of **‘4042** (i.p.) in neuropathic pain model in mice after SNI with mechanical allodynia (n = 5; two-way ANOVA; SNI x drug treatment interaction: $F(2, 24) = 0.5$, $P > 0.05$; SNI: $F(2, 24) = 51.8$, $P < 0.0001$; drug treatment: $F(1, 24) = 1.6$, $P > 0.05$; asterisks define individual group differences to vehicle control after Tukey’s multiple comparisons post-hoc test correction). Data presented are normalized to pre-SNI baseline measurements. **C.** Effect of **‘4042** (i.p.) in neuropathic pain model in mice after SNI with mechanical allodynia (n = 5; two-way ANOVA; SNI x drug treatment interaction: $F(1, 16) = 0.1$, $P > 0.05$; SNI: $F(1, 16) = 9.6$, $P = 0.007$; drug treatment: $F(1, 16) = 0.1$, $P > 0.05$; asterisks define individual group differences to vehicle control after Tukey’s multiple comparisons post-hoc test correction). Data presented are normalized to post-SNI baseline measurements. **D.** Effect of **‘4042** (i.p.) in naïve (non-SNI) mice in the mechanical assay (all n = 5; two-tailed unpaired t-test, $t(8) = 2.17$, $P > 0.05$). **E.** Effect of **‘4042** (i.t.) in neuropathic pain model in mice after SNI with mechanical allodynia (n = 5; one-way ANOVA, $F(6, 28) = 4.2$, P = 0.004; asterisks define individual group differences to vehicle control after Dunnett’s multiple comparisons post-hoc test correction). Data presented are normalized to pre-SNI baseline measurements. **F.** Effect of **‘4042** (i.t.) in neuropathic pain model in mice after SNI with mechanical allodynia (n = 5; one-way ANOVA, $F(7, 32) = 3.8$, $P = 0.004$; asterisks define individual group differences to vehicle control after Dunnett’s multiple comparisons post-hoc test correction). Data presented are normalized to post-SNI baseline measurements. **G.** Chemical hyperalgesia test after spared nerve injury (all n = 5; **‘4042** vs. vehicle: multiple two-tailed unpaired t-tests, total: $t(8) = 4.6$, $P = 0.007$; paw withdrawal: $t(8) = 6.2$, $P = 0.001$; paw shake: $t(8) = 4.5$, $P = 0.007$; paw lick and jump: $P > 0.05$; CP-55,940 vs. vehicle: multiple two-tailed unpaired t-tests, total: $t(8) = 9.3$, $P < 0.0001$; paw withdrawal: $t(8) = 5.9$, $P = 0.002$; paw shake, paw lick, and jump: $P > 0.05$, asterisks define differences to vehicle control after the Holm-Šídák multiple comparisons post-hoc test correction; **‘1350** vs. vehicle: two-way ANOVA; behavior x dose interaction: $F(12, 80) = 8.2$, $P < 0.0001$; behavior: $F(4, 80) = 69.6$, $P <$ 0. 0001; dose: $F(3, 80) = 34.2$, $P < 0.0001$; asterisks define individual group differences to vehicle control after Dunnett’s multiple comparisons post-hoc test correction). **H.** Tail flick latency after co-treatment with the selective CB1 antagonist AM251 (all n = 5; one-way ANOVA, $F(2, 17) = 29.9$, $P < 0.0001$; asterisks define individual group differences to baseline control after Tukey’s multiple comparisons post-hoc test correction. **I.** Comparison of the effect of **‘4042** and CP-55,940 in wildtype (WT) versus CB2R knockout (KO) mice in the Hargreaves assay (all n = 5; two-way ANOVA; genotype x drug treatment interaction: $F(2, 24) = 0.5$, $P > 0.05$; genotype: $F(1, 24) = 1.6$, $P > 0.05$; drug treatment: $F(2, 24) = 13.8$, $P = 0.0001$; asterisks define individual group differences to baseline after Tukey’s multiple comparisons post-hoc test correction). **J.** Comparison of the effect of **‘4042** in wildtype (WT) versus CB2R knockout (KO) mice in the Tail Flick assay (all n = 5; two-way ANOVA; genotype x drug treatment interaction: $F(1, 16) = 2.2$, $P > 0.05$; genotype: $F(1, 16) = 2.2$, $P > 0.05$; drug treatment: $F(1, 16) = 72.3$, $P < 0.0001$; asterisks define individual group differences to baseline after Šídák’s multiple comparisons post-hoc test correction). **K.** Withdrawal latency in the Hargreaves assay after co-treatment with the selective CB2R antagonist SR 144528 (1 mg/kg) (all n = 5; one-way ANOVA, $F(2, 17) = 6.6$, $P = 0.008$; asterisks define individual group differences to vehicle control after Tukey’s multiple comparisons post-hoc test correction). **L.** Mesh grip test of catalepsy at 1 hour post-dose. Comparison of CP-55,940 (n = 5-10; one-way ANOVA, $F(3, 26) = 10.3$, $P = 0.0001$), haloperidol (n = 5; two-tailed unpaired t-test, $t(8) = 3.5$, $P = 0.009$), **‘4042** (n = 5; one-way ANOVA, $F(3, 16) = 3.0$, $P > 0.05$) and **‘1350** (n = 5-10; one-way ANOVA, $F(3, 26) = 1.8$, $P > 0.05$). Asterisks define differences between 1 mg/kg dose for each compound and respective vehicle control. Data at 30 min. timepoint are in **Fig. 6**. **M.** Comparison of morphine (n = 8; two-tailed unpaired t-test, $t(14) = 2.51$, $P = 0.03$) to CP-55,940 (n = 8; two-tailed unpaired t-test, $t(14) = 2.9$, $P = 0.01$) and **‘4042** (n = 8; two-tailed unpaired t-test, $t(14) = 0.005$, $P > 0.05$) in the Conditioned Place Preference (CPP) test. For all statistical tests: ns, not significant, *$P < 0.05$, **$P < 0.01$, ***$P < 0.001$, ****$P < 0.0001$. All data represent mean ± SEM of 5-10 animals.
